# The predictive value of baseline symptom score and the peripheral CD4CD8 double-positive T cells in patients with AECOPD

**DOI:** 10.1186/s12890-023-02751-7

**Published:** 2023-11-29

**Authors:** Shiyi He, Shiyu Wu, Tianwei Chen, Weina Huang, Aiping Yu, Chao Cao

**Affiliations:** 1 Department of Respiratory and Critical Care Medicine, Key Laboratory of Respiratory Disease of Ningbo, The First Affiliated Hospital of Ningbo University, Ningbo, China; 2 Health Science Center, Ningbo University, Ningbo, China; 3 Zhejiang University School of Medicine, Hangzhou, China; 4 Department of Nursing, The First Affiliated Hospital of Ningbo University, Ningbo, China

**Keywords:** Chronic obstructive pulmonary disease, Acute exacerbations, Autoimmunity, Prediction nomogram, CD4^+^CD8^+^ T cells, COPD assessment test

## Abstract

**Background:**

Accurate prediction of acute exacerbation helps select patients with chronic obstructive pulmonary disease (COPD) for individualized therapy. The potential of lymphocyte subsets to function as clinical predictive factors for acute exacerbations of chronic obstructive pulmonary disease (AECOPD) remains uncertain.

**Methods:**

In this single-center prospective cohort study with a 2-year follow-up, 137 patients aged 51 to 79 with AECOPD were enrolled. We examined the prognostic indicators of AECOPD by analyzing lymphocyte subsets and baseline symptom score. Furthermore, a predictive model was constructed to anticipate the occurrence of respiratory failure in patients experiencing AECOPD.

**Results:**

The COPD Assessment Test (CAT) score combined with home oxygen therapy and CD4^+^CD8^+^ T cells% to predict respiratory failure in AECOPD patients were the best (the area under the curves [AUC] = 0.77, 95% CI: 0.70–0.86, *P* < 0.0001, sensitivity: 60.4%, specificity: 86.8%). The nomogram model, the C index, calibration plot, decision curve analysis, and clinical impact curve all indicate the model’s good predictive performance. The observed decrease in the proportions of CD4^+^CD8^+^ T cells appears to be correlated with more unfavorable outcomes.

**Conclusions:**

The nomogram model, developed to forecast respiratory failure in patients with AECOPD, utilizing variables such as home oxygen therapy, CAT score, and CD4^+^CD8^+^ T cells%, demonstrated a high level of practicality in clinical settings. CD4^+^CD8^+^ T cells serve as a reliable and readily accessible predictor of AECOPD, exhibiting greater stability compared to other indices. It is less susceptible to subjective influences from patients or physicians. This model facilitated personalized estimations, enabling healthcare professionals to make informed decisions regarding preventive interventions.

**Supplementary Information:**

The online version contains supplementary material available at 10.1186/s12890-023-02751-7.

## Introduction

Before COVID-19 (coronavirus disease 2019), Chronic Obstructive Pulmonary Disease (COPD), characterized by airflow limitation, was the third leading cause of death in the worldwide pandemic [1]. Each year, almost half the patients with COPD have acute exacerbations beyond normal day-to-day symptoms that lead to a change in treatment or hospitalization, and a quarter have frequent (two or more) acute exacerbations [2, 3]. Driven by frequent exacerbations (≥ 2 events) of COPD, worse health status, hospital re-admission, and higher risk of death cause high healthcare costs and resource utilization [4].

COPD is a non-specific inflammatory disease, including innate and adaptive immunity, and macrophages, neutrophils, and T lymphocytes play important roles [5]. Infections caused by viruses or bacteria account for about 70 to 80 percent of COPD exacerbations. Both the modulating immune response to viral infection and the humoral immune response to new bacterial strains suggest the relevance of immune response to exacerbations of COPD [6–8]. The findings indicate that T lymphocytes, including CD4^+^ and CD8^+^ T cells, are essential in destroying lung tissues and airway obstruction. Th1, the primary type of CD4^+^ T lymphocytes elevating in lung parenchyma and airways, can secret inflammatory cytokines and chemokines to alveolar injuries [9, 10].

The findings from our previous study, which demonstrated a negative correlation between complement C3 levels and adverse outcomes in individuals with AECOPD, provide further evidence supporting the hypothesis that autoimmunity may play a role in the occurrence of exacerbations in COPD [11]. In this study, we propose the hypothesis that a particular subset of lymphocytes may serve as effectors in the advancement of autoimmune disorders.

## Methods

### Study design

This study was conducted as a single-center prospective cohort study with a follow-up period of 2 years, spanning from January 2020 to June 2022. The study received approval from the Research Ethics Committee at Ningbo First Hospital (No. 2016-R017). At baseline, age, gender, BMI, smoking index, duration of COPD, comorbidities (diabetes mellitus, coronary disease, stroke, hypertension), modified Medical Research Council (mMRC) score, exercise capacity score, St George's Respiratory Questionnaire (SGRQ) score, COPD Assessment Test (CAT) score, COPD-related exacerbation within the previous year and the history of home oxygen therapy, regular medication, oral glucocorticoid therapy, theophylline therapy, expectorants therapy, inhaled COPD therapy were documented. Laboratory parameters included the levels of lymphocyte subsets. The short-term outcomes were using NIV, respiratory failure, and systemic glucocorticoids. Participants were followed up every six months by telephone calls and computerized hospital records for collecting the long-term outcomes, including the data on hospital re-admission, exacerbation, and death. The severity of the exacerbations was defined by Anthonisen criteria [1]. All patients provided written informed consent before participating in the study, in accordance with the guidelines set forth by the Declaration of Helsinki. The participants were provided with detailed information regarding the study protocols and ethical considerations. The confidentiality of both the participants and the collected data was assured. The review authors will implement stringent data security measures to safeguard the data from any unauthorized access or tracking.

### Participants

We enrolled 137 patients aged 51 to 79 with AECOPD undergoing a treatment in Ningbo First Hospital. Before the commencement of the study, the corresponding author assessed the subjects' eligibility for the study and screened them. Inclusion criteria were: diagnosis of AECOPD, patients ˃18 years old. The clinical diagnosis of COPD was assessed by patients' medical history and spirometry (a post-bronchodilator ratio of forced expiratory volume in one second [FEV1]/forced vital capacity [FVC] < 0.7). We excluded subjects if they (1) could not complete the questionnaires or the test of lymphocyte subset, (2) had any other significant respiratory diseases such as bronchiectasis, interstitial lung disease, and lung cancer, (3) unstable psychiatric morbidity or exhibited cognitive impairment, (4) were younger than 18 years, (5) had a complicating or confounding condition.

### Data collection

Lymphocyte Subsets: In the morning, skilled nurses conducted the collection of blood samples and their subsequent transportation. Under standardized conditions, venous blood samples (2 ml) were drawn into K3EDTA anticoagulant-containing tubes and transported to the laboratory for the proportion of lymphocyte subsets, including T lymphocytes, CD4^+^ T cells, CD8^+^ T cells, CD4^+^CD8^+^ T cells, CD4^−^CD8^−^ T cells, natural killer cells (NK cells), B lymphocytes populations by flow cytometry analysis. According to the “lyse no wash” protocol, 50 μl of peripheral blood was transferred into each BD TruCount tube (BD Biosciences, San Jose, CA, USA). And the corresponding fluorescence-labeled antibodies were added into a TruCount tube incubated under the dark for 15 min at room temperature. Erythrocytes were lysed by 450 μL BD FACS lysing buffer (BD Biosciences, San Jose, CA, USA). The samples were analyzed with the FACS Canto analyzer (BD Biosciences, San Jose, CA, USA) with BD FACSDiva software.

Questionnaires: The SGRQ (symptoms, activity, and psychosocial impact subscales) was used for assessing the patients’ health-related quality of life (HRQOL) [12, 13]. For grading the effect of perceived dyspnea, we used the mMRC, which consisted of five items about the impact of breathlessness on daily activities [13]. The CAT was used to measure the effect on health status [14]. The exercise capacity scale was employed to evaluate the level of intensity associated with activities performed in the course of daily living. This scale is delineated as follows: 0, signifying the absence of any limitations in activity; 1, denoting the capability to engage in simple work and leisurely walks without any constraints; 2, indicating the ability to stroll with some restrictions, yet still able to visit nearby supermarkets and vegetable markets; 3, representing the inability to venture beyond one's residence.

### Statistical analysis

The normality of distribution for quantitative data was evaluated using the Shapiro–Wilk test in order to obtain descriptive data. Mean ± standard deviation (SD) was used to report normally distributed continuous variables, while median and interquartile range were utilized for non-normally distributed variables. Chi-square (χ2) tests were employed to analyze categorical variables. To compare two groups, the independent t-test was employed to analyze continuous variables that followed a normal distribution, while the Mann–Whitney U test was utilized for non-normally distributed variables. To compare groups, we utilized one-way ANOVA to analyze the within-group differences of normally distributed continuous variables, and the Kruskal–Wallis test was employed for non-normally distributed variables.

The variables with significant differences (*P* < 0.05) in the univariate analyses were included in the multivariate model. Multiple logistic regression was performed to analyze the different indicators of COPD exacerbation status. The odds ratio (OR), the 95% confidence intervals (CIs), and the p-value of each factor from the multivariate logistic regression models were estimated and presented. Receiver operating characteristic curve (ROC) analyses were performed to obtain the area under the curves (AUC). AUC was applied to compare the prediction ability. The determination of the cutoff value was accomplished through the utilization of ROC curve and the calculation of AUC.

The multivariate Cox regression models included the variables with significant differences (*P* < 0.05) in the univariate analysis. Unadjusted and adjusted hazard ratios (HRs), 95% CIs, and p-value of each factor from the Cox regression models were estimated and presented. A two-tailed value of *P* < 0.05 was considered to be significant in all statistical analyses. The nomogram was constructed using the R package “rms”. The objective of constructing the nomogram model was to forecast the occurrence of respiratory failure in patients with AECOPD, by incorporating variables such as home oxygen therapy, CAT score, and the percentage of CD4^+^CD8^+^ T cells%. The concordance index (C index), calibration plots, decision curve analysis (DCA), and clinical impact curves were used to assess the prediction model. The bootstrap resampling was used for internal validation [15]. In order to address the potential influence of outliers on the study's findings, the bootstrapping resampling technique was utilized to validate the predictive model. The bootstrap resampling technique was employed to generate 1,000 cohorts with replacement, randomly sampled from the original cohort. Statistical analysis was performed using SPSS version 19.0 (SPSS Inc., Chicago, IL, USA) and R software 4.1.2 (R Foundation for Statistical Computing).

## Results

### Patients

**Fig. 1:**
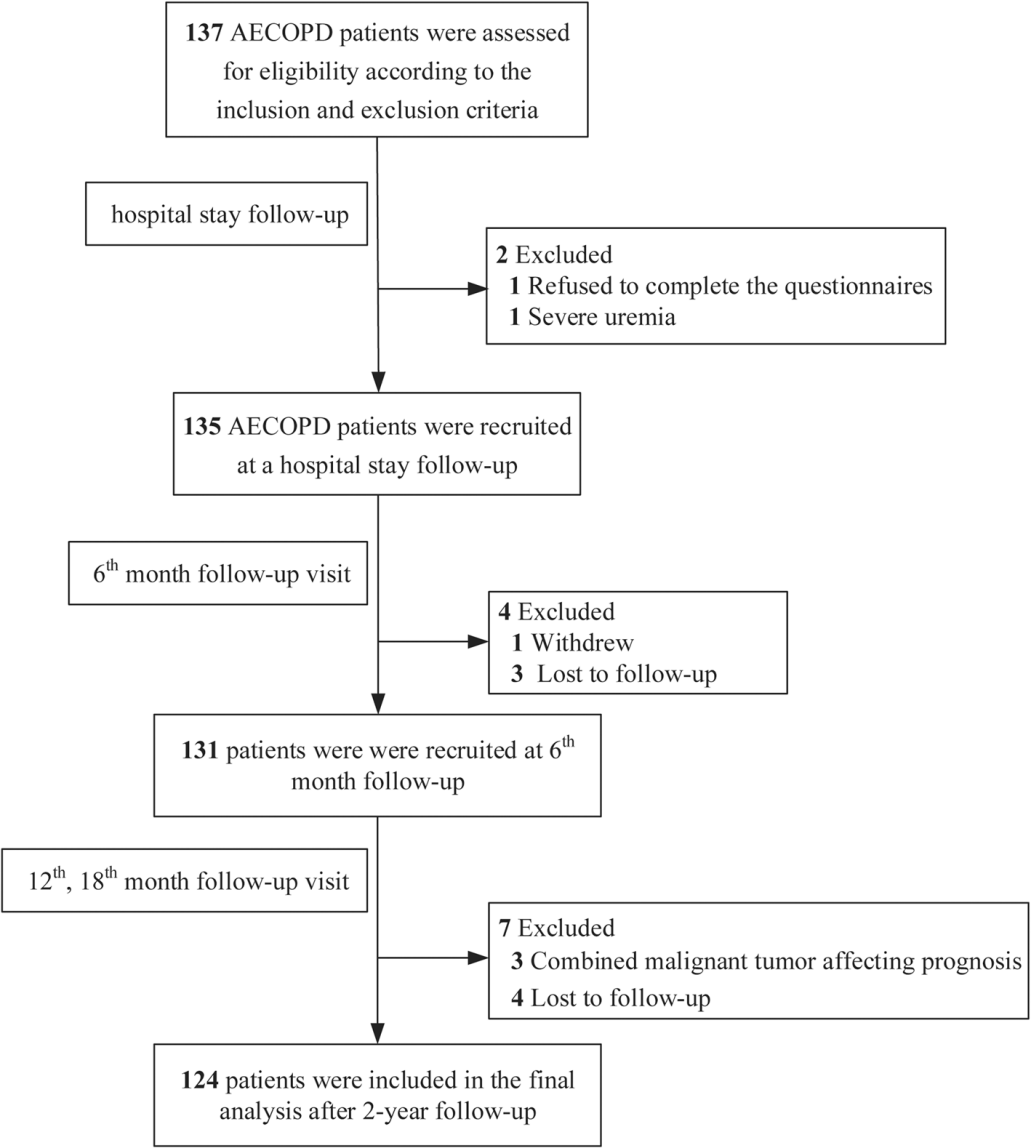
Participant flow diagram

Of the 2020 to 2021 patients screened, 137 were eligible, and 124 patients had two years of complete prospective follow-up data, including one patient who was excluded due to refusing to complete the questionnaires, one patient with severe uremia, one due to withdrawal, three with malignant tumor affecting prognosis, and seven with loss to follow-up. Finally, we recruited 124 patients for the final analysis, and the details regarding screening and follow-up are provided in Fig. 1.

### Respiratory failure

The respiratory failure group had a higher SGRQ score (*P* = 0.006) and exercise capacity score (*P* = 0.0002) than the no respiratory failure groups, with a more significant proportion of patients having previous home oxygen therapy (*P* = 0.002) (Table 1). Compared with patients without respiratory failure, patients with respiratory failure were likelier to have more COPD-related exacerbations (*P* = 0.019) within the previous year before the study entry. In Table 2, lymphocyte subsets analysis showed that the respiratory failure group had significantly lower CD4^+^CD8^+^ T cells percentage versus the no respiratory failure group (median 0.68 [IQR 0.38–1.11] vs 0.89 [0.62–1.54], *P* = 0.02).

**Table 1:** Study cohort characteristics are stratified by respiratory failure in patients with acute exacerbation of COPD

Characteristic	Respiratory Failure (*N* = 48)		No Respiratory Failure (*N* = 76)		*P* Value
	**N**	**Value**	**N**	**Value**	
Age^a^	48	66.0(62.3,72.8)	76	70.0(63.3,73.0)	0.13
Female gender^b^	39	81.3%	65	85.5%	0.62
BMI^a^	48	20.8(17.3,22.4)	76	21.2(18.4,22.9)	0.18
Smoking Index ^a^	48	600(0,975)	76	675(363,1350)	0.32
Duration of COPD^a^	48	13(6,29)	76	10(6,20)	0.31
Comorbidities^b^	24	50.0%	41	53.9%	0.71
Diabetes mellitus^b^	7	14.6%	7	9.2%	0.39
Coronary disease^b^	3	6.3%	5	6.6%	1.00
Stroke^b^	0	0%	3	3.9%	0.28
Hypertension^b^	21	43.8%	37	48.7%	0.71
Home oxygen therapy^b^	26	54.2%	20	26.3%	0.002
Regular medication^b^	29	60.4%	40	52.6%	0.46
Oral glucocorticoid therapy^b^	3	6.3%	2	2.6%	0.37
Theophylline therapy^b^	1	2.1%	1	1.3%	1.00
Expectorants therapy^b^	16	33.3%	18	23.7%	0.30
Inhaled COPD therapy^b^		-	-	-	0.25
No inhaled treatment^b^	5	10.4%	15	19.7%	
LAMA^b^	3	6.3%	6	7.9%	
ICS + LABA^b^	5	10.4%	15	19.7%	
LABA + LAMA^b^	7	14.6%	9	11.8%	
ICS + LABA + LAMA^b^	28	58.3%	31	40.8%	
SGRQ score^a^	48	50.5(35.1,74.1)	76	37.2(25.4,58.5)	0.003
mMRC score^a^	48	2(1,3)	76	1(1,3)	0.006
Exercise capacity score^a^	48	2(1,2)	76	1(1,2)	0.003
CAT score^a^	48	26(21,32)	76	19(13,26)	0.0002
COPD-related exacerbation within the previous year^a^	48	1(0,2)	76	0(0,1)	0.019

*COPD* chronic obstructive pulmonary disease, *BMI* body mass index, *LAMA* Long-acting muscarinic antagonists, *LABA* long-acting beta2-agonists, *ICS* inhaled corticosteroids, *SGRQ* St George's respiratory questionnaire, *mMRC* modified medical research council, *CAT* chronic obstructive pulmonary disease assessment test

Data are expressed as

^a^median; 25–75th percentile

^b^data are expressed as %. *P* values: to evaluate the differences between the two groups, an independent t-test was used to analyze the normally distributed continuous variables; the Mann–Whitney U test was used to analyze the non-normally distributed variables; and Chi-square (χ2) tests were used to analyze the categorical variables

**Table 2:** Effects of lymphocyte subsets on respiratory failure in patients with acute exacerbation of COPD

Variables	Respiratory Failure (*N* = 48)		No Respiratory Failure (*N* = 76)		*P* Value
	**N**	**Value**	**N**	**Value**	
T lymphocytes%^a^	48	65.40 ± 10.86	76	67.15 ± 12.58	0.43
CD4^+^ T cells%^a^	48	37.69 ± 11.28	76	40.21 ± 10.30	0.20
CD8^+^ T cells%^b^	48	25.4(17.9,32.3)	76	25.4(19.5,31.6)	0.98
CD4^+^CD8^+^ T cells%^b^	48	0.68(0.38,1.11)	76	0.89(0.62,1.54)	0.02
CD4^−^CD8^−^ T cells%^b^	48	1.68(0.66,3.75)	76	1.65(0.69,3.16)	0.82
NK cells%^b^	48	21.0(11.7,27.7)	76	18.0(11.2,28.8)	0.77
T4/T8 ratio%^b^	48	1.56(0.98,2.03)	76	1.70(1.18,2.38)	0.30
B lymphocytes%^b^	48	11.4(7.8,17.2)	76	11.7(6.8,15.7)	0.74

*COPD* chronic obstructive pulmonary disease, *NK cells* natural killer cells

Data are expressed as

^a^mean ± SD

^b^median; 25–75th percentile. *P* values: to evaluate the differences between the two groups, an independent t-test was used to analyze the normally distributed continuous variables; the Mann–Whitney U test was used to analyze the non-normally distributed variables

**Fig. 2:**
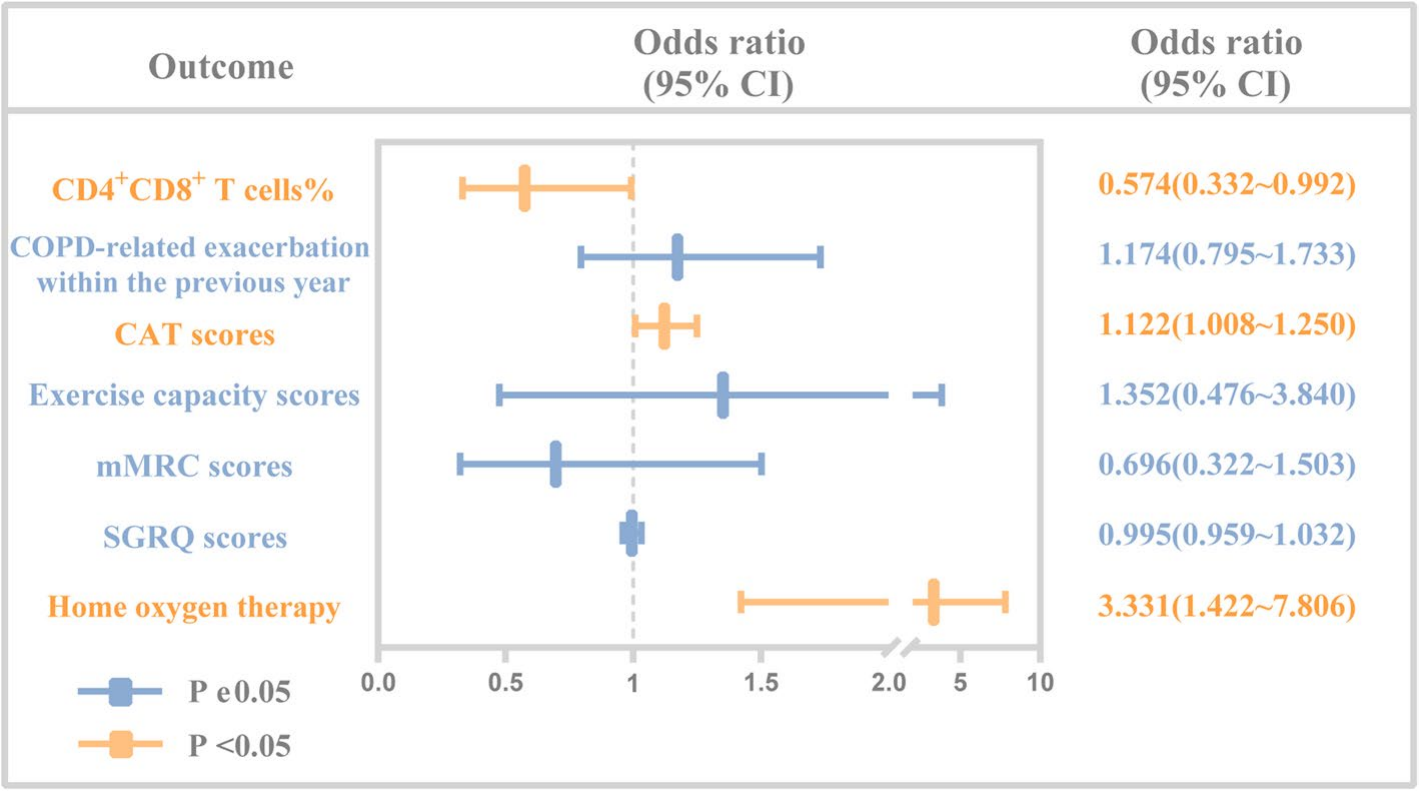
Odds ratios of the independent variables to predict respiratory failure in patients with acute exacerbation of COPD. Binary logistic regression data showing the OR with 95% CI for home oxygen therapy SGRQ score, mMRC score, Exercise capacity score, CAT score, COPD-related exacerbation within the previous year, CD4^+^CD8^+^ T cells% to predict respiratory failure in patients with acute exacerbation of COPD. COPD, chronic obstructive pulmonary disease; OR, odds ratio; CI, confidence interval; SGRQ, St George's respiratory questionnaire; mMRC, modified medical research council; CAT, chronic obstructive pulmonary disease assessment test

**Fig. 3:**
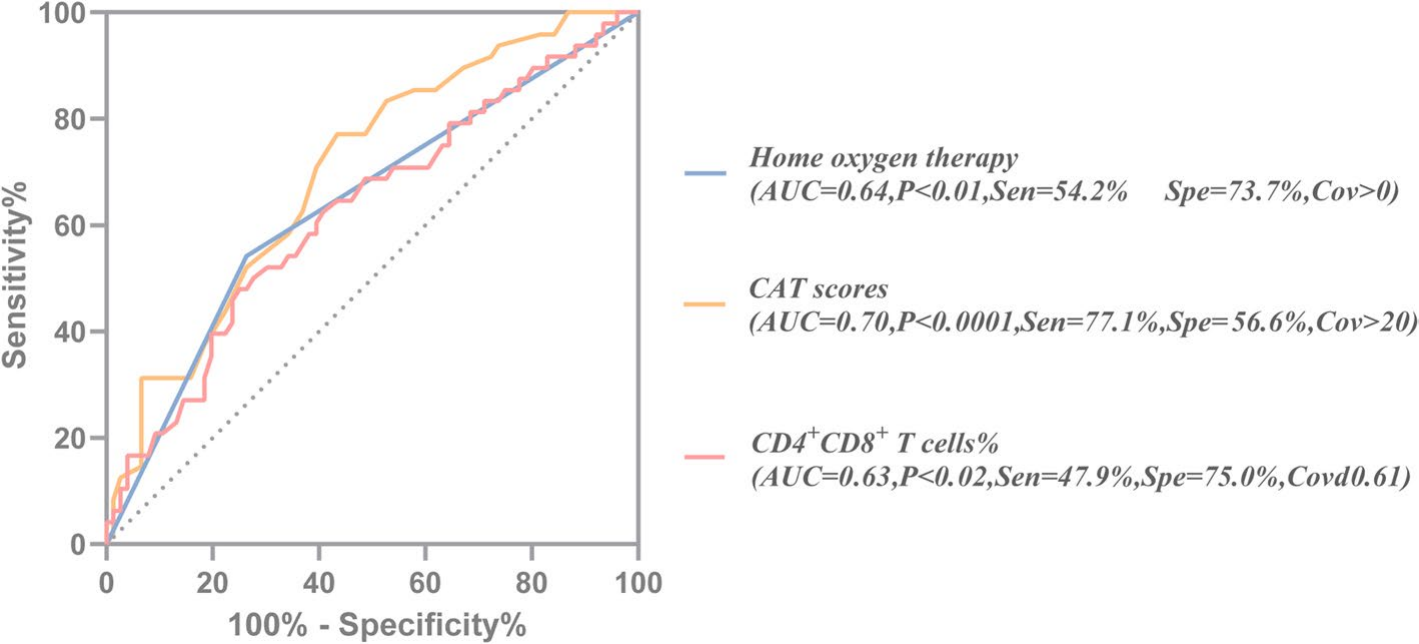
ROC curve analysis of CD4^+^CD8^+^ T cells%, CAT score, and home oxygen therapy for predicting respiratory failure in patients with acute exacerbation of COPD. COPD, chronic obstructive pulmonary disease; AUC, the area under the curve; Sen, sensitivity; Spe, specificity; Cov, cut-off value; ROC, receiver operating characteristic; CAT, chronic obstructive pulmonary disease assessment test

**Fig. 4:**
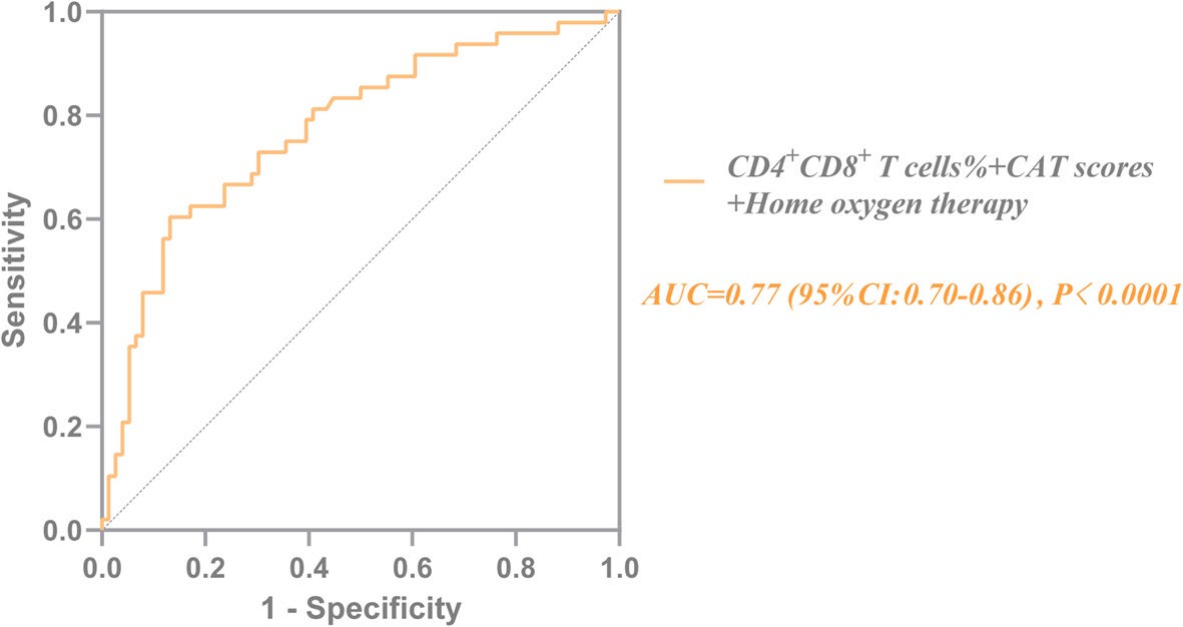
ROC curve for risk factors of combined CD4^+^CD8^+^ T cells%, CAT score, and home oxygen therapy for predicting respiratory failure in patients with acute exacerbation of COPD. The sensitivity and specificity were 60.4% and 86.8%, respectively. COPD, chronic obstructive pulmonary disease; AUC, the area under the curve; Sen, sensitivity; Spe, specificity; Cov, cut-off value; ROC, receiver operating characteristic; CAT, chronic obstructive pulmonary disease assessment test

By binary logistic regression, we accessed the risk factors associated with respiratory failure. The results showed that patients had a previous home oxygen therapy (OR: 3.331; 95% CI: 1.422–7.806; *P* = 0.006), higher CAT score (OR: 1.122; 95% CI: 1.008–1.250; *P* = 0.036), and lower CD4^+^CD8^+^ T cells percentage (OR: 0.574; 95% CI: 0.332–0.992; *P* = 0.047) were associated with a higher risk of respiratory failure (Table 3, Fig. 2). ROC analyses for home oxygen therapy prediction of respiratory failure showed a moderate, although significant, prediction (AUC = 0.64, *P* < 0.01); ROC analyses for CD4^+^CD8^+^ T cells% showed similar results (AUC = 0.63, *P* < 0.02, sensitivity: 47.9%, specificity: 75.0%, Cov ≤ 0.61) (Fig. 3). In terms of predicted ability, all three parameters were suitable, but the CAT score was the best predictor (AUC = 0.70, *P* < 0.0001, Cov > 20) (Fig. 3). Moreover, we found that the CAT score combined with home oxygen therapy and CD4^+^CD8^+^ T cells% to predict respiratory failure in AECOPD patients were the best (AUC = 0.77, 95% CI: 0.70–0.86, *P* < 0.0001, sensitivity: 60.4%, specificity: 86.8%) (Fig. 4).

**Table 3:** Multivariate analysis for respiratory failure in patients with acute exacerbation of COPD

Variables	B	*P*	OR	95% C.I. for OR	
				**Min**	**Max**
Home oxygen therapy	1.203	0.006	3.331	1.422	7.806
SGRQ score	-0.005	0.782	0.995	0.959	1.032
mMRC score	-0.363	0.356	0.696	0.322	1.503
Exercise capacity score	0.302	0.571	1.352	0.476	3.840
CAT score	0.115	0.036	1.122	1.008	1.250
COPD-related exacerbation within the previous year	0.160	0.421	1.174	0.795	1.733
CD4^+^CD8^+^ T cells%	-0.556	0.047	0.574	0.332	0.992
Constant	-2.681	0.001	0.068		

Binary logistic regression was used

*OR* odds ratio, *CI* confidence interval, *Max* maximum, *Min* minimum, *COPD* chronic obstructive pulmonary disease, *SGRQ* St George's respiratory questionnaire, *mMRC* modified medical research council, *CAT* chronic obstructive pulmonary disease assessment test

**Fig. 5:**
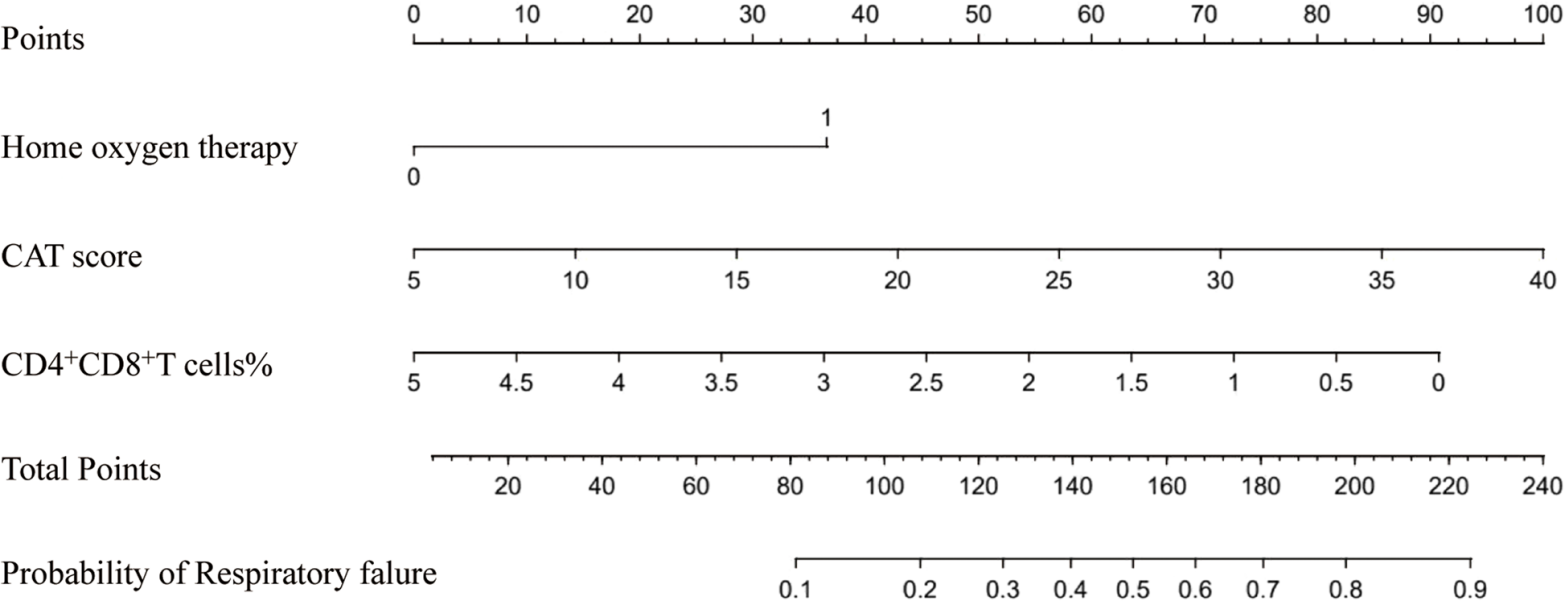
The nomogram for respiratory failure in patients with acute exacerbation of COPD. COPD, chronic obstructive pulmonary disease; CAT, chronic obstructive pulmonary disease assessment test

**Fig. 6:**
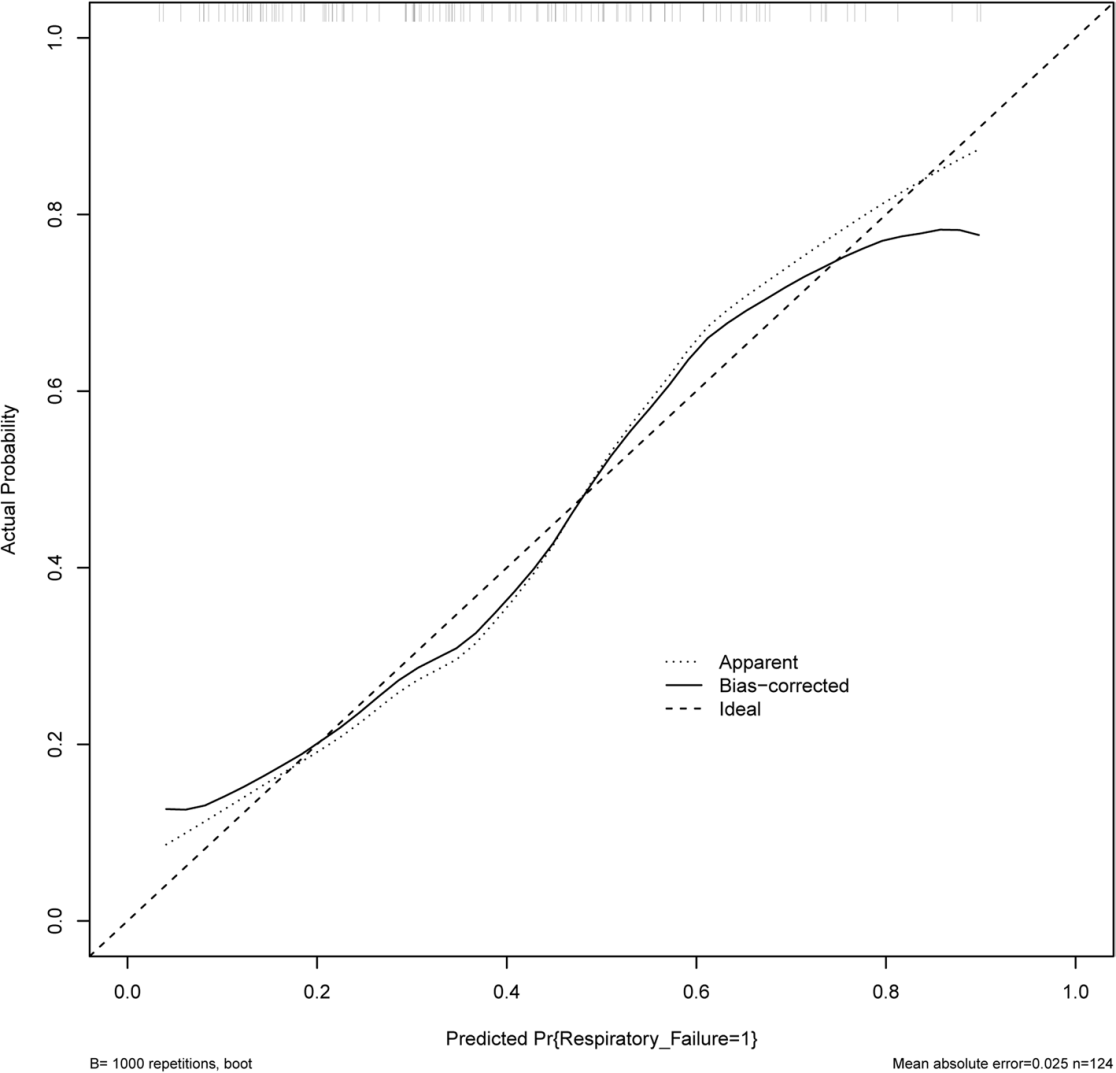
Calibration curve of the nomogram

**Fig. 7:**
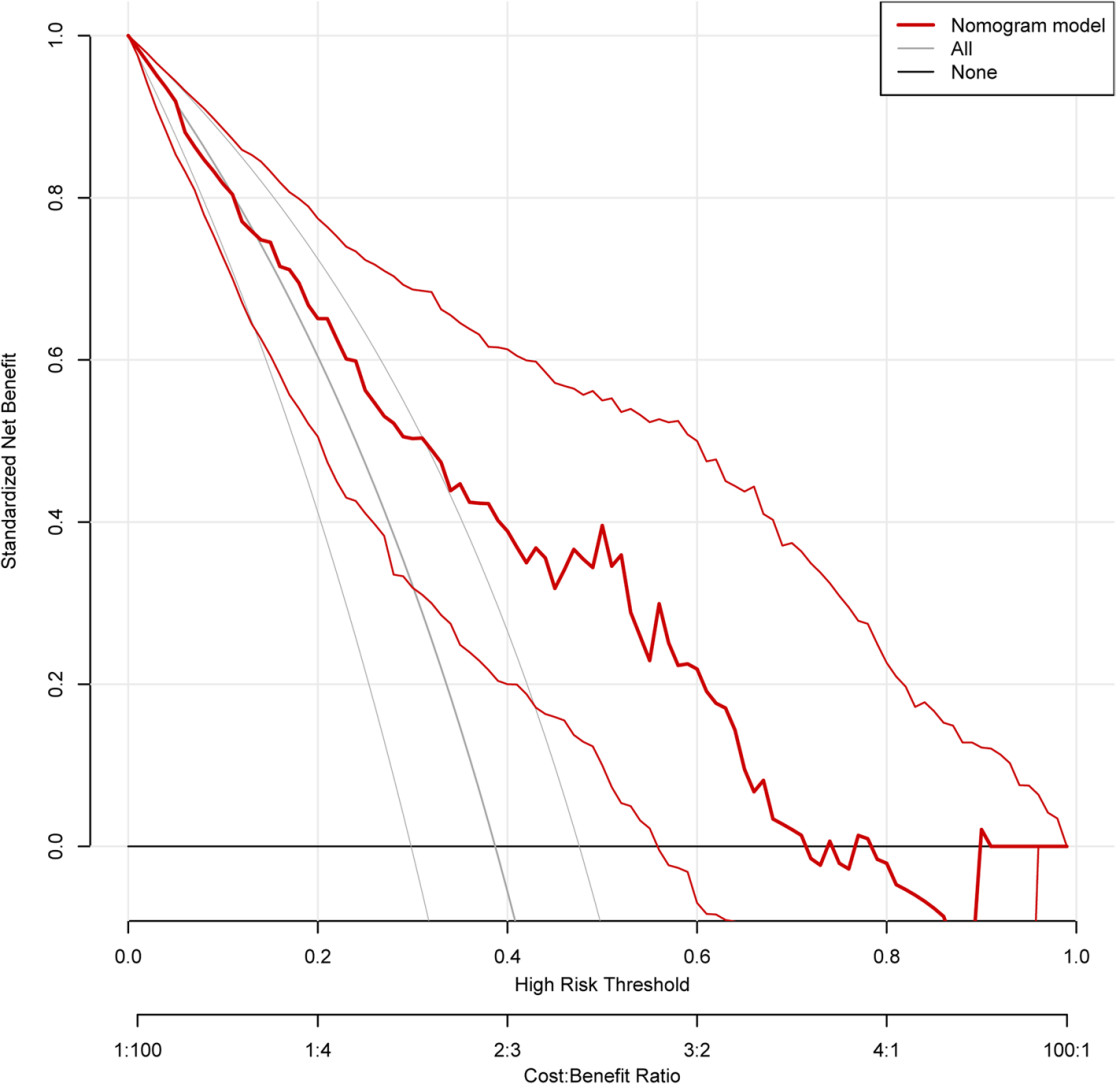
The decision curve analysis of the nomogram

**Fig. 8:**
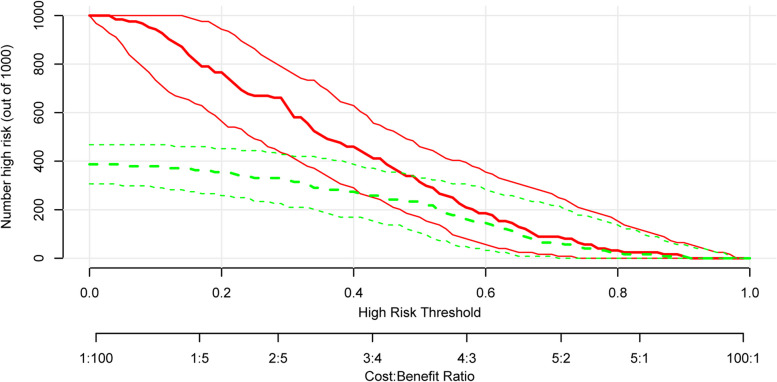
The clinical impact curve

The nomogram model’s construction aimed to predict respiratory failure in patients with AECOPD, utilizing variables such as home oxygen therapy, CAT score, and the percentage of CD4^+^CD8^+^ T cells (Fig. 5). The C index was 0.772 in the training cohort. We assessed the performance of this prediction model by calibration plot (Fig. 6), and the mean absolute error was 0.025, showing good agreement. We evaluated the efficacy of DCA (Fig. 7), and applying the nomogram to guide clinical decision-making had a pretty excellent net benefit. To further predict respiratory failure for a population of 1000, we plotted the clinical impact curve (Fig. 8). After bootstrap resampling (*n* = 200 samples), the C index was 0.773 (95%CI: 0.767–0.779), which remained materially unchanged. The C index, calibration plot, decision curve analysis (DCA), and clinical impact curve collectively demonstrate that the model exhibited a commendable level of predictive efficacy.

### Systemic glucocorticoids

We also compared the demographic and clinical characteristics of patients with AECOPD with systemic glucocorticoids versus no systemic glucocorticoids (Table S1). The systemic glucocorticoid group had higher SGRQ (*P* = 0.001), mMRC (*P* = 0.005), exercise capacity (*P* = 0.04), and CAT (*P* = 0.0001) scores than the no systemic glucocorticoid groups, and a more significant proportion of patients having a previous home oxygen therapy (*P* = 0.009), more COPD-related exacerbations within the last year (*P* = 0.0001), more regular medication (*P* = 0.02). Inhaled COPD therapy significantly differed between the systemic and no systemic glucocorticoid groups (*P* = 0.002, Table S1). There were no significant differences in all lymphocyte subsets between systemic glucocorticoid and no systemic glucocorticoid groups (all *P* > 0.05, Table S2). After further binary logistic regression, no parameter was independently associated with systemic glucocorticoid use in AECOPD patients (all *P* > 0.05, Table S3).

### Noninvasive ventilation

Patients with NIV had significantly higher SGRQ scores (*P* = 0.001), mMRC scores (*P* < 0.0001), exercise capacity scores (*P* = 0.0002), CAT scores (*P* = 0.0003), more COPD-related exacerbations within the previous year (*P* = 0.01), and a more significant proportion of patients having prior home oxygen therapy (*P* = 0.02) (Table S4). AECOPD patients without NIV had higher proportions of CD4^+^CD8^+^ T cells than NIV (0.6 versus 0.9, *P* = 0.007). However, the two groups had no significant differences in other lymphocyte subsets (all *P* > 0.05, Table S5). We accessed the risk factors associated with NIV in AECOPD patients; no parameter was independently associated with NIV in AECOPD patients (all *P* > 0.05, Table S6).

### COPD exacerbation status

Severe Exacerbators presented more symptoms (mMRC score, median 2 points [IQR 1–3] vs 1 point [1-3], *P* < 0.05), worse behavior of quality of life (SGRQ score, median 46.2 points [IQR 36.9–73.1] vs 33.3 points [24.7–60.0], *P* < 0.001), greater disease severity (CAT score, mean 24.3 points [SD 7.4] vs 21 points [SD 7.7], *P* < 0.05), a more significant proportion of patients having prior home oxygen therapy (*P* < 0.05), and COPD-related exacerbation within the previous year (*P* < 0.001) than no exacerbators (Table 4). Regarding clinical variables, T lymphocytes% was higher in mild (median 72.0 [IQR 63.6–78.9] vs 63.7 [IQR 55.6–72.8], *P* < 0.05) and severe exacerbators (median 70.1 points [IQR 62.3–75.9] vs 63.7 [IQR 55.6–72.8], *P* < 0.05) than in no exacerbators, and the NK cells% was lower in mild exacerbators than in no exacerbators (median 12.2 [IQR 6.1–22.0] vs 24.2 [IQR 13.4–33.5], *P* < 0.05) (Table 5). However, only home oxygen therapy (*P* = 0.04), SGRQ score (*P* = 0.04), mMRC score (*P* = 0.04), CAT score (*P* = 0.049), and COPD-related exacerbation within the previous year (*P* = 0.002) were significant univariable factors of AECOPD. The multiple logistic regression evaluating the relationship between them and the risk of exacerbation is summarized in Table 6. COPD-related exacerbation within the previous year (OR: 2.71; 95% CI: 1.12–6.57; *P* = 0.03) and the history of home oxygen therapy (OR: 1.71; 95% CI: 1.10–2.65; *P* = 0.02) were significant risk factors of severe exacerbations (Table 6).

**Table 4:** Study cohort characteristics are stratified by COPD exacerbation status with a 2-year follow-up

Characteristic	No Exacerbators (*N* = 53)		Mild Exacerbators (*N* = 15)		Moderate Exacerbators (*N* = 7)		Severe Exacerbators (*N* = 49)		*P* Value
	**N**	**Value**	**N**	**Value**	**N**	**Value**	**N**	**Value**	
Age^b^	53	70(63,75)	15	66(59,71)	7	71(65,72)	49	67(63,73)	0.17
Female gender^c^	43	81.1%	14	93.3%	4	57.1%	43	87.8%	0.14
BMI^b^	53	21.2(18.4,22.6)	15	21.5(19.1,23.7)	7	18.6(17.9,24.2)	49	20.8(17.7,22.8)	0.62
Smoking Index ^b^	53	800(25,1550)	15	420(260,800)	7	0(0,1600)	49	600(375,950)	0.38
Duration of COPD^b^	53	10(6,25)	15	15(10,15)	7	7(4,15)	49	10(6,25)	0.66
Comorbidities^c^	30	56.6%	8	53.3%	4	57.1%	23	46.9%	0.79
Diabetes mellitus^c^	9	17.0%	1	6.7%	0	0%	4	8.2%	0.33
Coronary disease^c^	4	7.5%	2	13.3%	0	0%	2	4.1%	0.53
Stroke^c^	1	1.9%	0	0%	1	14.3%	1	2.0%	0.20
Hypertension ^c^	28	52.8%	7	46.7%	3	42.9%	20	40.8%	0.68
Home oxygen therapy^c^	14	26.4%	6	40.0%	1	14.3%	25	51.0%^b^	0.04
Regular medication^c^	26	49.1%	7	46.7%	4	57.1%	32	65.3%	0.35
Oral glucocorticoid therapy^c^	3	5.7%	1	6.7%	0	0%	1	2.0%	0.70
Theophylline therapy^c^	0	0%	0	0%	0	0%	2	4.1%	0.38
Expectorants therapy^c^	13	24.5%	6	40.0%	1	14.3%	14	28.6%	0.56
Inhaled COPD therapy^c^		-	-	-	-	-	-	-	0.05
No inhaled treatment^c^	9	17.0%	5	33.3%	3	42.9%	3	6.1%	
LAMA^c^	3	5.7%	2	13.3%	0	0%	4	8.2%	
ICS + LABA^c^	12	22.6%	1	6.7%	2	28.6%	5	10.2%	
LABA + LAMA^c^	8	15.1%	0	0%	1	14.3%	7	14.3%	
ICS + LABA + LAMA^c^	21	39.6%	7	46.7%	1	14.3%	30	61.2%	
SGRQ score^b^	53	33.3(24.7,60.0)	15	43.8(26.1,63.3)	7	41.5(26.1,51.0)	49	46.2(36.9,73.1)^d^	0.04
mMRC score^b^	53	1(1,3)	15	1(1,3)	7	1(0,2)	49	2(1,3)^*e*^	0.04
Exercise capacity score^b^	53	1(1,2)	15	1(1,2)	7	1(1,2)	49	2(1,2)^*e*^	0.05
CAT score^**a**^	53	21.0 ± 7.7	15	20.1 ± 8.6	7	18.1 ± 7.9	49	24.3 ± 7.4^*e*^	0.049
COPD-related exacerbation within the previous year^b^	53	0(0,1)	15	0(0,1)	7	0(0,2)	49	1(0,2)^d^	0.002

*COPD* chronic obstructive pulmonary disease, *BMI* body mass index, *LAMA* Long-acting muscarinic antagonists, *LABA* long-acting beta2-agonists, *ICS* inhaled corticosteroids, *SGRQ* St George's respiratory questionnaire, *mMRC* modified medical research council, *CAT* chronic obstructive pulmonary disease assessment test

Data are expressed as

^a^mean ± SD

^b^median; 25–75th percentile

^c^data are expressed as %. *P* values: One-way ANOVA was used to analyze the normally distributed continuous variables of within-group difference between the COPD exacerbation status. The Kruskal–Wallis test was used for the non-normally distributed variables. Chi-square (χ2) tests were used to analyze the categorical variables

^d^*P* < 0.001

^*e*^*P* < 0.05 for comparison with individuals with COPD but no exacerbation history

**Table 5:** Effects of lymphocyte subsets on different COPD exacerbation statuses with a 2-year follow-up

Variables	No Exacerbators (*N* = 53)		Mild Exacerbators (*N* = 15)		Moderate Exacerbators (*N* = 7)		Severe Exacerbators (*N* = 49)		*P* Value
	**N**	**Value**	**N**	**Value**	**N**	**Value**	**N**	**Value**	
T lymphocytes%^b^	53	63.7(55.6,72.8)	15	72.0(63.6,78.9)^d^	7	71.2(44.2,74.2)	49	70.1(62.3,75.9)^d^	0.09
CD4^+^ T cells%^a^	53	37.5 ± 11.1	15	43.5 ± 8.5	7	37.1 ± 11.6	49	40.0 ± 10.6	0.23
CD8^+^ T cells%^b^	53	23.4(17.5,32.1)	15	26.4(19.9,31.4)	7	19.9(14.5,41.2)	49	25.9(19.2,32.1)	0.90
CD4^+^CD8^+^ T cells%^b^	53	0.85(0.56,1.58)	15	1.02(0.53,1.24)	7	0.97(0.17,1.76)	49	0.71(0.38,1.07)	0.22
CD4^−^CD8^−^ T cells%^b^	53	1.66(0.43,3.39)	15	2.41(1.35,3.97)	7	1.76(0.71,2.83)	49	1.48(0.66,3.37)	0.65
NK cells%^b^	53	24.2(13.4,33.5)	15	12.2(6.1,22.0)^d^	7	15.5(11.1,47.5)	49	18.4(10.1,26.3)	0.07
T4/T8 ratio%^b^	53	1.62(1.03,2.43)	15	1.71(1.25,2.04)	7	1.92(0.81,2.40)	49	1.59(1.07,2.12)	0.92
B lymphocytes%^b^	53	11.6(6.1,16.2)	15	13.0(8.8,16.9)	7	11.5(8.4,15.8)	49	11.4(7.3,17.0)	0.79

*COPD* chronic obstructive pulmonary disease, *NK cells* natural killer cells

Data are expressed as

^a^mean ± SD

^b^median; 25–75th percentile. *P* values: One-way ANOVA was used to analyze the normally distributed continuous variables of within-group difference between the COPD exacerbation status. The Kruskal–Wallis test was used for the non-normally distributed variables

^c^*P* < 0.001

^d^*P* < 0.05 for comparison with individuals with COPD but no exacerbation history

**Table 6:** Multivariate analysis for different COPD exacerbation statuses with a 2-year follow-up

Variables	Degree of exacerbation					
	**Mild Exacerbators**		**Moderate Exacerbators**		**Severe Exacerbators**	
	**Odds Ratio (95% CI)**	***P* value**	**Odds Ratio (95% CI)**	***P* value**	**Odds Ratio (95% CI)**	***P* value**
Home oxygen therapy	2.12(0.60,7.48)	0.24	0.55(0.06,5.45)	0.61	2.71(1.12,6.57)	0.03
SGRQ score	1.04(0.99,1.10)	0.13	1.05(0.98,1.13)	0.15	1.01(0.97,1.05)	0.66
mMRC score	0.99(0.41,2.38)	0.98	0.44(0.11,1.81)	0.26	1.13(0.61,2.08)	0.70
CAT score	0.89(0.77,1.03)	0.12	0.93(0.76,1.12)	0.43	0.99(0.89,1.10)	0.81
COPD-related exacerbation within the previous year	0.80(0.36,1.76)	0.58	1.43(0.61,3.32)	0.41	1.71(1.10,2.65)	0.02

Multiple logistic regression was used

*OR* odds ratio, *CI* confidence interval, *Max* maximum, *Min* minimum, *COPD* chronic obstructive pulmonary disease, *SGRQ* St George's respiratory questionnaire, *mMRC* modified medical research council, *CAT* chronic obstructive pulmonary disease assessment test

### Hospital re-admission

We compared patients’ demographic and clinical characteristics with hospital re-admission versus no re-admission in Table 7. The hospital re-admission group had a higher SGRQ score (*P* = 0.005), mMRC score (*P* = 0.005), exercise capacity score (*P* = 0.008), and CAT score (*P* = 0.01) than the no re-admission group, a more significant proportion of patients having a previous home oxygen therapy (*P* = 0.01), more COPD-related exacerbation within the last year (*P* = 0.0002). Inhaled COPD therapy also significantly differed between the two groups (*P* = 0.04). Regarding clinical variables, the group that experienced re-admission exhibited a statistically significant decrease in the percentage of CD4^+^CD8^+^ T cells compared to the group that did not experience re-admission (median 0.71 [IQR 0.38–1.07] vs 0.91 [0.53–1.48], *P* = 0.04) (Table 8). By binary logistic regression, we accessed the risk factors associated with re-admission. The results showed that only COPD-related exacerbation within the previous year (OR: 1.67; 95% CI: 1.09–2.55; *P* = 0.02) was associated with a higher risk of respiratory failure (Table 9).

**Table 7:** Study cohort characteristics stratified by COPD re‑admission with a 2-year follow-up

Characteristic	Re‑admission (*N* = 49)		No Re‑admission (*N* = 75)		*P* Value
	**N**	**Value**	**N**	**Value**	
Age^a^	49	67(63,73)	75	70(63,73)	0.31
Female gender^b^	43	87.8%	61	81.3%	0.46
BMI^a^	49	20.8(17.7,22.8)	75	21.2(18.4,22.8)	0.68
Smoking Index ^a^	49	600(375,950)	75	600(0,1350)	0.95
Duration of COPD^a^	49	10(6,25)	75	13(6,20)	0.93
Comorbidities^b^	23	46.9%	42	56.0%	0.36
Diabetes mellitus^b^	4	8.2%	10	13.3%	0.56
Coronary disease^b^	2	4.1%	6	8.0%	0.48
Stroke^b^	1	2.0%	2	2.7%	1.00
Hypertension^b^	20	40.8%	38	50.7%	0.36
Home oxygen therapy^b^	25	51.0%	21	28.0%	0.01
Regular medication^b^	32	65.3%	37	49.3%	0.10
Oral glucocorticoid therapy^b^	1	2.0%	4	5.3%	0.65
Theophylline therapy^b^	2	4.1%	0	0%	0.15
Expectorants therapy^b^	14	28.6%	20	26.7%	0.84
Inhaled COPD therapy^b^	-	-	-	-	0.04
No inhaled treatment^b^	3	6.1%	17	22.7%	
LAMA^b^	4	8.2%	5	6.7%	
ICS + LABA^b^	5	10.2%	15	20.0%	
LABA + LAMA^b^	7	14.3%	9	12.0%	
ICS + LABA + LAMA^b^	30	61.2%	29	38.7%	
SGRQ score^a^	49	46.2(36.9,73.1)	75	34.9(25.2,60.0)	0.005
mMRC score^a^	49	2(1,3)	75	1(1,3)	0.005
Exercise capacity score^a^	49	2(1,2)	75	1(1,2)	0.008
CAT score^a^	49	24(19,29)	75	20(13,27)	0.01
COPD-related exacerbation within the previous year^a^	49	1(0,2)	75	0(0,1)	0.0002

*COPD* chronic obstructive pulmonary disease, *BMI* body mass index, *LAMA* Long-acting muscarinic antagonists, *LABA* long-acting beta2-agonists, *ICS* inhaled corticosteroids, *SGRQ* St George's respiratory questionnaire, *mMRC* modified medical research council, *CAT* chronic obstructive pulmonary disease assessment test

Data are expressed as

^a^median; 25–75th percentile

^b^data are expressed as %

*P* values: to evaluate the differences between the two groups, an independent t-test was used to analyze the normally distributed continuous variables; the Mann–Whitney U test was used to analyze the non-normally distributed variables; and Chi-square (χ2) tests were used to analyze the categorical variables

**Table 8:** Effects of lymphocyte subsets on COPD re‑admission with a 2-year follow-up

Variables	Re‑admission (*N* = 49)		No Re‑admission (*N* = 75)		*P* Value
	**N**	**Value**	**N**	**Value**	
T lymphocytes%^b^	49	70.1(62.3,75.9)	75	64.7(57.8,74.1)	0.13
CD4^+^ T cells%^a^	49	40.03 ± 10.59	75	38.71 ± 10.84	0.50
CD8^+^ T cells%^b^	49	25.9(19.2,32.1)	75	24.6(18.1,32.1)	0.70
CD4^+^CD8^+^ T cells%^b^	49	0.71(0.38,1.07)	75	0.91(0.53,1.48)	0.04
CD4^−^CD8^−^ T cells%^b^	49	1.48(0.66,3.37)	75	1.75(0.71,3.30)	0.55
NK cells%^b^	49	18.4(10.1,26.3)	75	19.2(11.6,29.9)	0.43
T4/T8 ratio%^b^	49	1.59(1.07,2.12)	75	1.66(1.17,2.40)	0.94
B lymphocytes%^b^	49	11.4(7.3,17)	75	11.7(7.5,16.3)	0.83

*COPD* chronic obstructive pulmonary disease, *NK* cells, natural killer cells

Data are expressed as

^a^mean ± SD

^b^median; 25–75th percentile

*P* values: to evaluate the differences between the two groups, an independent t-test was used to analyze the normally distributed continuous variables; the Mann–Whitney U test was used to analyze the non-normally distributed variables

**Table 9:** Multivariate analysis for COPD re‑admission with a 2-year follow-up

Variables	B	*P*	OR	95% C.I. for OR	
				**Min**	**Max**
Home oxygen therapy	0.80	0.07	2.22	0.92	5.37
SGRQ score	-0.006	0.75	0.99	0.96	1.03
mMRC score	0.15	0.71	1.16	0.53	2.57
Inhaled COPD therapy	-	0.40	-	-	-
No inhaled treatment	-	-	1	-	-
LAMA	1.07	0.29	2.92	0.40	21.01
ICS + LABA	0.53	0.54	1.70	0.31	9.22
LABA + LAMA	1.46	0.10	4.31	0.77	24.10
ICS + LABA + LAMA	1.20	0.11	3.32	0.77	14.20
Exercise capacity score	0.01	0.98	1.01	0.34	2.93
CAT score	0.02	0.71	1.02	0.91	1.14
COPD-related exacerbation within the previous year	0.51	0.02	1.67	1.09	2.55
CD4^+^CD8^+^ T cells%	-0.45	0.10	0.64	0.37	1.08
Constant	-2.18	0.03	0.11		

Binary logistic regression was used

*OR* odds ratio, *CI* confidence interval, *Max* maximum, *Min* minimum, *COPD* chronic obstructive pulmonary disease, *SGRQ* St George's respiratory questionnaire, *mMRC* modified medical research council, *CAT* chronic obstructive pulmonary disease assessment test, *LAMA* Long-acting muscarinic antagonists, *LABA* long-acting beta2-agonists, *ICS* inhaled corticosteroids

### Frequent exacerbation

The demographic and clinical characteristics of frequent and no frequent exacerbators were shown in Table S7. Only COPD-related exacerbation within the previous year significantly differed between the two groups (*P* = 0.01). No significant differences were recorded between the two groups in other demographic, clinical characteristics, or lymphocyte subsets (all *P* > 0.05, Tables S7 and S8). After further binary logistic regression, neither COPD-related exacerbation within the previous year was not independently associated with frequent exacerbation (OR: 1.38; 95% CI: 0.98–1.94; *P* = 0.07, Table S9).

### Time to hospital re‑admission

As the time-to-event data, we analyzed the time to hospital re‑admission using univariate and multivariate Cox regression analyses. No statistically significant difference was observed in the demographic and clinical characteristics during the univariate analysis (all *P* > 0.05, Table S10). Repeating these analyses using lymphocyte subsets produced similar results (all *P* > 0.05, Table S11). The time to hospital re‑admission was not independently predicted by any available covariates (all *P* > 0.05, Table S12).

## Discussion

In this prospective, longitudinal, observational trial, the CAT score, home oxygen therapy, and CD4^+^CD8^+^ T cells% were significant predictors of respiratory failure in AECOPD patients. These results were consistent with previously reported findings that the abnormal T-lymphocytes may contribute to airflow limitation among patients with COPD, especially small airway disease [16].

In clinical practice, more objective indicators are required for individualized therapy. Therefore, this study explored the association between lymphocyte subsets and acute exacerbation for enriching clinical trials [17].

One interesting finding was that lower proportions of CD4^+^CD8^+^ T cells seem sociated with worse outcomes, including respiratory failure, re-admission, and NIV. Similar results are also seen in other studies. Several studies have reported a reduction in the concentration of CD4^+^ and CD8^+^ T cells in peripheral blood during acute exacerbations of chronic obstructive pulmonary disease [18, 19]. CD4 + CD8 + T cells are implicated in various normal and pathological conditions, encompassing autoimmune diseases and cancer [20]. However, there is a dearth of research exploring the functional roles of CD4^+^CD8^+^ T cells in AECOPD. Consequently, additional investigations are imperative to validate the pertinent mechanisms implicated. Though, in this study, CD4^+^CD8^+^ T cells% was not the independent predictor of exacerbation status, frequent exacerbation, or hospital re-admission, the associations between CD4^+^CD8^+^ T cells% and worse outcomes might suggest that this specific subset of lymphocytes may play a role as effectors in the progression of autoimmune disorders.

Considering that using the nomogram in detecting a respiratory failure in AECOPD patients to direct clinical management was rapid and cost-effective, we developed this prediction model incorporating variables such as home oxygen therapy, CAT score, and CD4^+^CD8^+^ T cells percentage. As a statistical modeling method, the nomogram incorporating the impact of diverse clinical parameters could comprehensively evaluate by calculating the score. In this research, this prediction model could identify respiratory failure in AECOPD patients with a comparatively good prediction ability of this model (C index = 0.772). Moreover, the C index, calibration plot, DCA, and clinical impact curve used to assess the clinical utilities and prognostic accuracies indicated that the model had a good predictive performance. These data support screening the CAT score, home oxygen therapy, and CD4^+^CD8^+^ T cells% in AECOPD patients to identify respiratory failure early, which could stabilize the patient's condition and benefit the numerous clinical outcomes in AECOPD patients.

Although several studies have reported that the factors associated with exacerbations and readmission to the hospital, including a history of gastroesophageal reflux or heartburn, elevated white-cell count, poorer health status, dyspnea, using long-term oxygen therapy, and a history of COPD-related exacerbation within the previous year, researchers widely recognized the history of COPD-related exacerbation was the strongest predictor of future exacerbation [3, 21]. Sang Do and colleagues also reported that the history of one exacerbation in the past year and the history of frequent exacerbations (≥ 2 events) were critical predictors for future exacerbations [22]. To confirm the associations reported previously, at baseline, the enrollment of our study included comorbidities, SGRQ score, mMRC score, CAT score, COPD-related exacerbation within the previous year, and the history of different therapies. Consistent with this view, we found a history of COPD-related exacerbation predicts re‑admission and severe exacerbation. Furthermore, the history of home oxygen therapy is an independent risk factor associated with respiratory failure in AECOPD patients. This is perhaps unsurprising given the severity of the disease according to the CAT score in the AECOPD cohort enrolled at baseline. The higher the CAT score, the greater the need for home oxygen therapy.

Some limitations of this study should be acknowledged. First, this study was a single-center prospective cohort study. It included a small sample size that only recruited 124 patients; therefore, the results might not be generalizable. In the future, we plan to conduct a multicenter study with a larger sample size to enhance the generalizability of the results. Second, as a purely observational study without specific interventions, the study might not assign causality between lymphocyte subsets and outcomes. Further investigation utilizing various methodologies and longitudinal designs is imperative in order to ascertain causal relationships. Third, we established a prediction model for respiratory failure in one cohort of small enrollment. We used the bootstrap resampling for internal validation but lacked external cohort verification. In the future, we may establish another cohort with more patients to validate our model. Finally, although telephone calls and computerized hospital records collected the exacerbation data, the portion of exacerbation data from the patient’s recall might underestimate the exacerbation rates. A dual verification process has been implemented to mitigate errors, whereby patients' medical records from electronic health records are cross-referenced with telephone follow-ups.

Although the above limitations, as a study evaluating the relationship between lymphocyte subsets and exacerbations, these findings in this single-center prospective cohort of AECOPD patients might have important implications for the recommended use of CAT score, home oxygen therapy, and CD4^+^CD8^+^ T cells% as predictive biomarkers to guide individualized respiratory failure therapies.

## Conclusions

The CAT score, home oxygen therapy, and CD4^+^CD8^+^ T cells% were identified as significant predictors of respiratory failure in AECOPD patients. Additionally, the integration of these factors showed the highest efficacy in predicting respiratory failure (AUC = 0.77, 95% CI: 0.70–0.86, *P* < 0.0001, sensitivity: 60.4%, specificity: 86.8%). This prognostic model has the potential to be refined for personalized forecasting of AECOPD. Providing timely clinical treatment and intervention to patients at high risk of deterioration can alleviate their suffering and economic burden to some extent. We acknowledge that our study is constrained by its single-center design and sample size, thereby potentially limiting the generalizability of the results.

### Supplementary Information

**Additional file 1:** **Table S1.** Study cohort characteristics are stratified by systemic glucocorticoids in patients with acute exacerbation of COPD. **Table S2.** Effects of lymphocyte subsets on systemic glucocorticoids in patients with acute exacerbation of COPD. **Table S3.** Multivariate analysis for systemic glucocorticoids in patients with acute exacerbation of COPD. **Table S4.** Study cohort characteristics are stratified by noninvasive ventilation in patients with acute exacerbation of COPD. **Table S5.** Effects of lymphocyte subsets on noninvasive ventilation in patients with acute exacerbation of COPD. **Table S6.** Multivariate analysis for noninvasive ventilation in patients with acute exacerbation of COPD. **Table S7.** Study cohort characteristics are stratified by frequent exacerbation with a 2-year follow-up. **Table S8.** Effects of lymphocyte subsets on frequent exacerbation with a 2-year follow-up. **Table S9.** Multivariate analysis for frequent exacerbation with a 2-year follow-up. **Table S10.** An unadjusted cox regression model including study cohort characteristics to predict the time to hospital re‑admission. **Table S11.** An unadjusted Cox regression model including lymphocyte subsets to predict the time to hospital re‑admission. **Table S12.** Adjusted cox regression model to predict the time to hospital re‑admission

## Data Availability

The datasets used and analyzed during the current study are available from the corresponding author upon reasonable request.

## Abbreviations

AECOPD: Acute exacerbations of chronic obstructive pulmonary disease
COVID-19: Coronavirus disease 2019
COPD: Chronic Obstructive Pulmonary Disease
NIV: Noninvasive ventilation
SGRQ: St George's Respiratory Questionnaire
mMRC: Modified Medical Research Council
CAT: COPD Assessment Test
FEV1: Forced expiratory volume in one second
FVC: Forced vital capacity
HRQOL: Health-related quality of life
MCID: Minimum clinically important difference
SD: Standard deviation
OR: Odds ratio
CI: Confidence interval
ROC: Receiver operating characteristic curve
AUC: Area under the curves
Cov: Cut-off value
HR: Hazard ratio
C index: Concordance index
DCA: Decision curve analysis
NK cells: Natural killer cells
