# Supplementary material for: The predictive value of baseline symptom score and the peripheral CD4CD8 double-positive T cells in patients with AECOPD
Source: BMC Pulm Med. 2023 Nov 29;23:478. doi: 10.1186/s12890-023-02751-7 (PMC10685522; doi:10.1186/s12890-023-02751-7)
Supplement: Supplementary file 1 — Additional file 1: Table S1. Study cohort characteristics are stratified by systemic glucocorticoids in patients with acute exacerbation of COPD. Table S2. Effects of lymphocyte subsets on systemic glucocorticoids in patients with acute exacerbation of COPD. Table S3. Multivariate analysis for systemic glucocorticoids in patients with acute exacerbation of COPD. Table S4. Study cohort characteristics are stratified by noninvasive ventilation in patients with acute exacerbation of COPD. Table S5. Effects of lymphocyte subsets on noninvasive ventilation in patients with acute exacerbation of COPD. Table S6. Multivariate analysis for noninvasive ventilation in patients with acute exacerbation of COPD. Table S7. Study cohort characteristics are stratified by frequent exacerbation with a 2-year follow-up. Table S8. Effects of lymphocyte subsets on frequent exacerbation with a 2-year follow-up. Table S9. Multivariate analysis for frequent exacerbation with a 2-year follow-up. Table S10. An unadjusted cox regression model including study cohort characteristics to predict the time to hospital re‑admission. Table S11. An unadjusted Cox regression model including lymphocyte subsets to predict the time to hospital re‑admission. Table S12. Adjusted cox regression model to predict the time to hospital re‑admission [file 12890_2023_2751_MOESM1_ESM.docx]

**Table S1.** Study cohort characteristics are stratified by systemic glucocorticoids in patients with acute exacerbation of COPD

| **Characteristic** | **Systemic Glucocorticoids (N=67)** | | **No Systemic Glucocorticoids**  **(N=57)** | | ***P* Value** |
| --- | --- | --- | --- | --- | --- |
|  | **N** | **Value** | **N** | **Value** |  |
| Age**^#^** | 67 | 68(63,73) | 57 | 69(63,72) | 0.39 |
| Female genderˆ | 56 | 83.6% | 48 | 84.2% | 1.00 |
| BMI**^#^** | 67 | 20.8(17.7,23.2) | 57 | 21.2(18.4,22.5) | 0.68 |
| Smoking Index **^#^** | 67 | 800(360,1050) | 57 | 600(0,1200) | 0.52 |
| Duration of COPD**^#^** | 67 | 13(6,25) | 57 | 10(5,23) | 0.20 |
| Comorbiditiesˆ | 36 | 53.7% | 29 | 50.9% | 0.86 |
| Diabetes mellitusˆ | 11 | 16.4% | 3 | 5.3% | 0.09 |
| Coronary diseaseˆ | 3 | 4.5% | 5 | 62.5% | 0.47 |
| Strokeˆ | 0 | 0% | 3 | 8.8% | 0.09 |
| Hypertensionˆ | 31 | 46.3% | 27 | 47.4% | 1.00 |
| Home oxygen therapyˆ | 32 | 47.8% | 14 | 24.6% | 0.009 |
| Regular medicationˆ | 44 | 65.7% | 25 | 43.9% | 0.02 |
| Oral glucocorticoid therapyˆ | 4 | 6.0% | 1 | 1.8% | 0.37 |
| Theophylline therapyˆ | 1 | 1.5% | 1 | 1.8% | 1.00 |
| Expectorants therapyˆ | 21 | 31.3% | 13 | 22.8% | 0.32 |
| Inhaled COPD therapyˆ |  | - | - | - | 0.002 |
| No inhaled treatmentˆ | 7 | 10.4% | 13 | 22.8% |  |
| LAMAˆ | 2 | 3.0% | 7 | 12.3% |  |
| ICS+LABAˆ | 11 | 16.4% | 9 | 15.8% |  |
| LABA+LAMAˆ | 5 | 7.5% | 11 | 19.3% |  |
| ICS+LABA+LAMAˆ | 42 | 62.7% | 17 | 29.8% |  |
| SGRQ score^#^ | 67 | 48.6(33.3,74.4) | 57 | 36.3(24.9,48.6) | 0.001 |
| mMRC score^#^ | 67 | 2(1,3) | 57 | 1(1,3) | 0.005 |
| Exercise capacity score^#^ | 67 | 2(1,2) | 57 | 1(1,2) | 0.04 |
| CAT score^#^ | 67 | 25(18,31) | 57 | 19(13,25) | 0.0001 |
| COPD-related exacerbation within the previous year^#^ | 67 | 1(0,2) | 57 | 0(0,1) | 0.0001 |

Data are expressed as ^#^, median; 25–75th percentile; ˆ, data are expressed as %. *P* values: to evaluate the differences between the two groups, an independent t-test was used to analyze the normally distributed continuous variables; the Mann-Whitney U test was used to analyze the non-normally distributed variables; and Chi-square (χ2) tests were used to analyze the categorical variables. COPD, chronic obstructive pulmonary disease; BMI, body mass index; LAMA, Long-acting muscarinic antagonists; LABA, long-acting beta2-agonists; ICS, inhaled corticosteroids; SGRQ, St George's respiratory questionnaire; mMRC, modified medical research council; CAT, chronic obstructive pulmonary disease assessment test.

**Table S2.** Effects of lymphocyte subsets on systemic glucocorticoids in patients with acute exacerbation of COPD

| **Variables** | **Systemic Glucocorticoids (N=67)** | | **No Systemic Glucocorticoids**  **(N=57)** | | ***P* Value** |
| --- | --- | --- | --- | --- | --- |
|  | **N** | **Value** | **N** | **Value** |  |
| T lymphocytes%**^#^** | 67 | 65.5(58.0,74.6) | 57 | 70.0(59.9,78.5) | 0.15 |
| CD4^+^ T cells%***** | 67 | 38.6±11.2 | 57 | 40.0±10.2 | 0.46 |
| CD8^+^ T cells%**^#^** | 67 | 24.1(18.1,31.4) | 57 | 26.8(19.9,32.3) | 0.23 |
| CD4^+^CD8^+^ T cells%**^#^** | 67 | 0.77(0.52,1.20) | 57 | 0.85(0.53,1.62) | 0.37 |
| CD4^-^CD8^-^ T cells%**^#^** | 67 | 1.66(0.70,3.19) | 57 | 1.75(0.66,3.39) | 0.86 |
| NK cells%**^#^** | 67 | 19.9(11.6,27.9) | 57 | 17.6(11.1,32.0) | 0.80 |
| T4/T8 ratio%**^#^** | 67 | 1.63(1.20,2.14) | 57 | 1.66(1.04,2.25) | 0.96 |
| B lymphocytes%**^#^** | 67 | 12.5(8.2,17.3) | 57 | 11.3(6.8,15.1) | 0.19 |

Data are expressed as *, mean ± SD; ^#^, median; 25–75th percentile. *P* values: to evaluate the differences between the two groups, an independent t-test was used to analyze the normally distributed continuous variables; the Mann-Whitney U test was used to analyze the non-normally distributed variables. COPD, chronic obstructive pulmonary disease; NK cells, natural killer cells.

**Table S3.** Multivariate analysis for systemic glucocorticoids in patients with acute exacerbation of COPD

| **Variables** | **B** | ***P*** | **OR** | **95% C.I. for OR** | |
| --- | --- | --- | --- | --- | --- |
|  |  |  |  | **Min** | **Max** |
| Regular medication | 0.51 | 0.33 | 1.67 | 0.59 | 4.73 |
| Inhaled COPD therapy | - | 0.14 | - | - | - |
| No inhaled treatmentˆ | - | - | 1 | - | - |
| LAMAˆ | -1.59 | 0.15 | 0.20 | 0.02 | 1.78 |
| ICS+LABAˆ | 0.41 | 0.58 | 1.51 | 0.35 | 6.50 |
| LABA+LAMAˆ | -0.80 | 0.40 | 0.45 | 0.07 | 2.85 |
| ICS+LABA+LAMAˆ | 0.45 | 0.53 | 1.57 | 0.39 | 6.31 |
| Home oxygen therapy | 0.55 | 0.26 | 1.73 | 0.66 | 4.52 |
| SGRQ score | 0.01 | 0.56 | 1.01 | 0.98 | 1.05 |
| mMRC score | 0.19 | 0.26 | 1.21 | 0.51 | 2.88 |
| Exercise capacity score | -0.78 | 0.67 | 0.46 | 0.14 | 1.48 |
| CAT score | 0.09 | 0.19 | 1.09 | 0.98 | 1.22 |
| COPD-related exacerbation within the previous year | 0.48 | 0.10 | 1.62 | 1.00 | 2.61 |
| Constant | -2.42 | 0.005 | 0.09 |  |  |

Binary logistic regression was used. OR, odds ratio; CI, confidence interval; Max, maximum; Min, minimum; COPD, chronic obstructive pulmonary disease; SGRQ, St George's respiratory questionnaire; mMRC, modified medical research council; CAT, chronic obstructive pulmonary disease assessment test; LAMA, Long-acting muscarinic antagonists; LABA, long-acting beta2-agonists; ICS, inhaled corticosteroids.

**Table S4.** Study cohort characteristics are stratified by noninvasive ventilation in patients with acute exacerbation of COPD.

| **Characteristic** | **Noninvasive Ventilation**  **(N=33)** | | **No Noninvasive Ventilation**  **(N=91)** | | ***P* Value** |
| --- | --- | --- | --- | --- | --- |
|  | **N** | **Value** | **N** | **Value** |  |
| Age**^#^** | 33 | 66(63,72) | 91 | 69(63,73) | 0.31 |
| Female genderˆ | 28 | 84.8% | 76 | 83.5% | 1.00 |
| BMI**^#^** | 33 | 20.8(18.0,22.3) | 91 | 21.1(18.2,22.9) | 0.37 |
| Smoking Index**^#^** | 33 | 600(25,1025) | 91 | 750(200,1200) | 0.45 |
| Duration of COPD**^#^** | 33 | 12(7,28) | 91 | 10(6,20) | 0.39 |
| Comorbiditiesˆ | 17 | 51.5% | 48 | 52.7% | 1.00 |
| Diabetes mellitusˆ | 4 | 12.1% | 10 | 11.0% | 1.00 |
| Coronary diseaseˆ | 2 | 6.1% | 6 | 6.6% | 1.00 |
| Strokeˆ | 0 | 0% | 3 | 3.3% | 0.56 |
| Hypertension ˆ | 16 | 48.5% | 42 | 46.2% | 0.84 |
| Home oxygen therapyˆ | 18 | 54.5% | 28 | 30.8% | 0.02 |
| Regular medicationˆ | 18 | 54.5% | 51 | 56.0% | 1.00 |
| Oral glucocorticoid therapyˆ | 2 | 6.1% | 3 | 3.3% | 0.61 |
| Theophylline therapyˆ | 1 | 3.0% | 1 | 1.1% | 0.46 |
| Expectorants therapyˆ | 13 | 39.4% | 21 | 23.1% | 0.11 |
| Inhaled COPD therapyˆ |  | - | - | - | 0.21 |
| No inhaled treatmentˆ | 2 | 6.1% | 18 | 19.8% |  |
| LAMAˆ | 1 | 3.0% | 8 | 8.8% |  |
| ICS+LABAˆ | 5 | 15.2% | 15 | 16.5% |  |
| LABA+LAMAˆ | 5 | 15.2% | 11 | 12.1% |  |
| ICS+LABA+LAMAˆ | 20 | 60.6% | 39 | 42.9% |  |
| SGRQ score^#^ | 33 | 56.9(38.4,78.4) | 91 | 37.2(25.2,60.0) | 0.001 |
| mMRC score^#^ | 33 | 3(2,3.5) | 91 | 1(1,3) | 0.00009 |
| Exercise capacity score^#^ | 33 | 2(1,2.5) | 91 | 1(1,2) | 0.0002 |
| CAT score^#^ | 33 | 26(22,32) | 91 | 20(14,26) | 0.0003 |
| COPD-related exacerbation within the previous year^#^ | 33 | 1(0,2) | 91 | 0(0,1) | 0.01 |

Data are expressed as ^#^, median; 25–75th percentile; ˆ, data are expressed as %. *P* values: to evaluate the differences between the two groups, an independent t-test was used to analyze the normally distributed continuous variables; the Mann-Whitney U test was used to analyze the non-normally distributed variables; and Chi-square (χ2) tests were used to analyze the categorical variables. COPD, chronic obstructive pulmonary disease; BMI, body mass index; LAMA, Long-acting muscarinic antagonists; LABA, long-acting beta2-agonists; ICS, inhaled corticosteroids; SGRQ, St George's respiratory questionnaire; mMRC, modified medical research council; CAT, chronic obstructive pulmonary disease assessment test.

**Table S5.** Effects of lymphocyte subsets on noninvasive ventilation in patients with acute exacerbation of COPD

| **Variables** | **Noninvasive Ventilation**  **(N=33)** | | **No Noninvasive Ventilation**  **(N=91)** | | ***P* Value** |
| --- | --- | --- | --- | --- | --- |
|  | **N** | **Value** | **N** | **Value** |  |
| T lymphocytes%***** | 33 | 63.4±11.3 | 91 | 67.6±12.0 | 0.08 |
| CD4^+^ T cells%***** | 33 | 36.4±10.4 | 91 | 40.3±10.7 | 0.08 |
| CD8^+^ T cells%**^#^** | 33 | 24.2(17.1,32.9) | 91 | 25.5(19.9,30.9) | 0.76 |
| CD4^+^CD8^+^ T cells%**^#^** | 33 | 0.6(0.4,1.0) | 91 | 0.9(0.6,1.5) | 0.007 |
| CD4^-^CD8^-^ T cells%**^#^** | 33 | 1.7(0.7,3.9) | 91 | 1.6(0.7,3.1) | 0.39 |
| NK cells%**^#^** | 33 | 22.0(13.5,32.3) | 91 | 17.9(11.1,27.8) | 0.16 |
| T4/T8 ratio%**^#^** | 33 | 1.5(0.9,2.0) | 91 | 1.7(1.2,2.3) | 0.35 |
| B lymphocytes%**^#^** | 33 | 11.3(7.2,18.8) | 91 | 11.7(7.6,15.8) | 0.98 |

Data are expressed as *, mean ± SD; #, median; 25–75th percentile. *P* values: to evaluate the differences between the two groups, an independent t-test was used to analyze the normally distributed continuous variables; the Mann-Whitney U test was used to analyze the non-normally distributed variables. COPD, chronic obstructive pulmonary disease; NK cells, natural killer cells.

**Table S6.** Multivariate analysis for noninvasive ventilation in patients with acute exacerbation of COPD

| **Variables** | **B** | ***P*** | **OR** | **95% C.I. for OR** | |
| --- | --- | --- | --- | --- | --- |
|  |  |  |  | **Min** | **Max** |
| Home oxygen therapy | 0.92 | 0.05 | 2.50 | 0.99 | 6.27 |
| SGRQ score | -0.004 | 0.86 | 1.00 | 0.96 | 1.04 |
| mMRC score | 0.35 | 0.42 | 1.41 | 0.61 | 3.30 |
| Exercise capacity score | 0.22 | 0.72 | 1.24 | 0.38 | 4.03 |
| CAT score | 0.03 | 0.67 | 1.03 | 0.91 | 1.16 |
| COPD-related exacerbation within the previous year | 0.19 | 0.36 | 1.21 | 0.81 | 1.82 |
| CD4^+^CD8^+^ T cells% | -0.63 | 0.05 | 0.53 | 0.28 | 1.01 |
| Constant | -2.48 | 0.007 | 0.08 |  |  |

Binary logistic regression was used. OR, odds ratio; CI, confidence interval; Max, maximum; Min, minimum; COPD, chronic obstructive pulmonary disease; SGRQ, St George's respiratory questionnaire; mMRC, modified medical research council; CAT, chronic obstructive pulmonary disease assessment test.

**Table S7.** Study cohort characteristics are stratified by frequent exacerbation with a 2-year follow-up.

| **Characteristic** | **Frequent Exacerbators**  **(N=29)** | | **No Frequent Exacerbators**  **(N=95)** | | ***P* Value** |
| --- | --- | --- | --- | --- | --- |
|  | **N** | **Value** | **N** | **Value** |  |
| Age**^#^** | 29 | 68.0(61.5,72.5) | 95 | 69.0(63.0,72.5) | 0.58 |
| Female genderˆ | 25 | 86.2% | 79 | 83.2% | 1.00 |
| BMI**^#^** | 29 | 20.8(17.7,23.3) | 95 | 20.9(18.4,22.7) | 0.79 |
| Smoking Index**^#^** | 29 | 800(350,1225) | 95 | 600(0,1200) | 0.54 |
| Duration of COPD**^#^** | 29 | 8(6,23) | 95 | 15(6,25) | 0.25 |
| Comorbiditiesˆ | 15 | 51.7% | 50 | 52.6% | 1.00 |
| Diabetes mellitusˆ | 1 | 3.4% | 13 | 13.7% | 0.19 |
| Coronary diseaseˆ | 1 | 3.4% | 7 | 7.4% | 0.68 |
| Strokeˆ | 0 | 0% | 3 | 3.2% | 1.00 |
| Hypertensionˆ | 14 | 48.3% | 44 | 46.3% | 1.00 |
| Home oxygen therapyˆ | 14 | 48.3% | 32 | 33.7% | 0.19 |
| Regular medicationˆ | 21 | 72.4% | 48 | 50.5% | 0.05 |
| Oral glucocorticoid therapyˆ | 1 | 3.4% | 4 | 4.2% | 1.00 |
| Theophylline therapyˆ | 2 | 6.9% | 0 | 0% | 0.05 |
| Expectorants therapyˆ | 9 | 31.0% | 25 | 26.3% | 0.64 |
| Inhaled COPD therapyˆ |  | - | - | - | 0.63 |
| No inhaled treatmentˆ | 3 | 10.3% | 17 | 17.9% |  |
| LAMAˆ | 2 | 6.9% | 7 | 7.4% |  |
| ICS+LABAˆ | 3 | 10.3% | 17 | 17.9% |  |
| LABA+LAMAˆ | 5 | 17.2% | 11 | 11.6% |  |
| ICS+LABA+LAMAˆ | 16 | 55.2% | 43 | 45.3% |  |
| SGRQ score^#^ | 29 | 42.8(36.4,61.8) | 95 | 39.5(26.1,64.8) | 0.34 |
| mMRC score^#^ | 29 | 2(1,3) | 95 | 1(1,3) | 0.26 |
| Exercise capacity score^#^ | 29 | 2(1,2) | 95 | 1(1,2) | 0.41 |
| CAT score^#^ | 29 | 23(16,26.5) | 95 | 22(15,28) | 0.86 |
| COPD-related exacerbation within the previous year^#^ | 29 | 1(0,2) | 95 | 0(0,1) | 0.01 |

Data are expressed as ^#^, median; 25–75th percentile; ˆ, data are expressed as %. *P* values: to evaluate the differences between the two groups, an independent t-test was used to analyze the normally distributed continuous variables; the Mann-Whitney U test was used to analyze the non-normally distributed variables; and Chi-square (χ2) tests were used to analyze the categorical variables. COPD, chronic obstructive pulmonary disease; BMI, body mass index; LAMA, Long-acting muscarinic antagonists; LABA, long-acting beta2-agonists; ICS, inhaled corticosteroids; SGRQ, St George's respiratory questionnaire; mMRC, modified medical research council; CAT, chronic obstructive pulmonary disease assessment test.

**Table S8.** Effects of lymphocyte subsets on frequent exacerbation with a 2-year follow-up

| **Variables** | **Frequent Exacerbators**  **(N=29)** | | **No Frequent Exacerbators**  **(N=95)** | | ***P* Value** |
| --- | --- | --- | --- | --- | --- |
|  | **N** | **Value** | **N** | **Value** |  |
| T lymphocytes%**^#^** | 29 | 70.4(64.4,75.3) | 95 | 66.2(57.8,74.9) | 0.18 |
| CD4^+^ T cells%***** | 29 | 40.0±8.8 | 95 | 39.0±11.3 | 0.67 |
| CD8^+^ T cells%**^#^** | 29 | 26.9(20.8,31.2) | 95 | 24.2(18.1,32.1) | 0.30 |
| CD4^+^CD8^+^ T cells%**^#^** | 29 | 0.75(0.59,0.95) | 95 | 0.85(0.51,1.40) | 0.48 |
| CD4^-^CD8^-^ T cells%**^#^** | 29 | 1.8(0.5,3.8) | 95 | 1.6(0.7,3.2) | 0.60 |
| NK cells%**^#^** | 29 | 18.6(11.5,26.3) | 95 | 18.4(11.5,29.1) | 0.46 |
| T4/T8 ratio%**^#^** | 29 | 1.59(1.13,1.94) | 95 | 1.66(1.10,2.43) | 0.96 |
| B lymphocytes%**^#^** | 29 | 11.0(5.8,14.4) | 95 | 12.1(7.9,16.9) | 0.16 |

Data are expressed as *, mean ± SD; ^#^, median; 25–75th percentile. *P* values: to evaluate the differences between the two groups, an independent t-test was used to analyze the normally distributed continuous variables; the Mann-Whitney U test was used to analyze the non-normally distributed variables. COPD, chronic obstructive pulmonary disease; NK cells, natural killer cells.

**Table S9.** Multivariate analysis for frequent exacerbation with a 2-year follow-up

| **Variables** | **B** | ***P*** | **OR** | **95% C.I. for OR** | |
| --- | --- | --- | --- | --- | --- |
|  |  |  |  | **Min** | **Max** |
| COPD-related exacerbation within the previous year | 0.32 | 0.07 | 1.38 | 0.98 | 1.94 |
| Constant | -1.48 | 0.000001 | 0.23 |  |  |

Binary logistic regression was used. OR, odds ratio; CI, confidence interval; Max, maximum; Min, minimum; COPD, chronic obstructive pulmonary disease.

**Table S10.** An unadjusted cox regression model including study cohort characteristics to predict the time to hospital re‑admission

| **Variables** | **N** | **Value** | **Unadjusted HR(95%CI)** | ***P* Value** |
| --- | --- | --- | --- | --- |
| Age**^#^** | 124 | 69(63,73) | 0.97(0.93,1.02) | 0.21 |
| Female genderˆ | 103 | 83.1% | 1.42(0.61,3.34) | 0.42 |
| BMI**^#^** | 124 | 20.9(18.3,22.7) | 0.95(0.88,1.03) | 0.21 |
| Smoking Index**^#^** | 124 | 600(105,1200) | 1.00(1.00,1.00) | 0.70 |
| Duration of COPD**^#^** | 124 | 11(6,24) | 1.00(0.97,1.02) | 0.94 |
| Comorbiditiesˆ | 65 | 52.4% | 0.74(0.42,1.30) | 0.30 |
| Diabetes mellitusˆ | 14 | 11.3% | 0.61(0.22,1.70) | 0.35 |
| Coronary diseaseˆ | 8 | 6.5% | 0.54(0.13,2.22) | 0.39 |
| Strokeˆ | 3 | 2.4% | 0.76(0.11,5.49) | 0.78 |
| Hypertension ˆ | 58 | 46.8% | 0.73(0.42,1.29) | 0.28 |
| Home oxygen therapyˆ | 46 | 37.1% | 0.95(0.54,1.67) | 0.01 |
| Regular medicationˆ | 69 | 55.6% | 1.60(0.89,2.88) | 0.12 |
| Oral glucocorticoid therapyˆ | 5 | 4.0% | 0.44(0.06,3.19) | 0.42 |
| Theophylline therapyˆ | 2 | 1.6% | 4.56(1.08,19.2) | 0.04 |
| Expectorants therapyˆ | 34 | 27.4% | 1.13(0.61,2.10) | 0.71 |
| Inhaled COPD therapyˆ |  | - | - | 0.11 |
| No inhaled treatmentˆ | 20 | 16.1% | Ref. |  |
| LAMAˆ | 9 | 7.3% | 3.44(0.77,15.3) | 0.11 |
| ICS+LABAˆ | 20 | 16.1% | 1.81(0.43,7.56) | 0.42 |
| LABA+LAMAˆ | 16 | 12.9% | 3.29(0.85,12.7) | 0.09 |
| ICS+LABA+LAMAˆ | 59 | 47.6% | 4.11(1.25,13.5) | 0.02 |
| SGRQ score^#^ | 124 | 40.7(26.8,64.4) | 1.02(1.01,1.03) | 0.007 |
| mMRC score^#^ | 124 | 1.5(1,3) | 1.34(1.08,1.66) | 0.008 |
| exercise capacity score^#^ | 124 | 1(1,2) | 1.07(0.77,1.48) | 0.012 |
| CAT score^#^ | 124 | 22(16,28) | 1.05(1.01,1.08) | 0.011 |
| COPD-related exacerbation within the previous year^#^ | 124 | 0(0,1) | 1.40(1.15,1.70) | 0.001 |

Univariate Cox regression models were used. N, number of participants; HR, hazard ratio; CI, confidence interval; COPD, chronic obstructive pulmonary disease; SGRQ, St George's respiratory questionnaire; mMRC, modified medical research council; CAT, chronic obstructive pulmonary disease assessment test; LAMA, Long-acting muscarinic antagonists; LABA, long-acting beta2-agonists; ICS, inhaled corticosteroids.

**Table S11.** An unadjusted Cox regression model including lymphocyte subsets to predict the time to hospital re‑admission

| **Variables** | **N** | **Value** | **Unadjusted HR(95%CI)** | ***P* Value** |
| --- | --- | --- | --- | --- |
| T lymphocytes%***** | 124 | 66.5±11.9 | 1.02(0.99,1.04) | 0.24 |
| CD4^+^ T cells%***** | 124 | 39.2±10.7 | 1.01(0.99,1.04) | 0.36 |
| CD8^+^ T cells%**^#^** | 124 | 25.4(18.5,32.1) | 1.01(0.98,1.03) | 0.74 |
| CD4^+^CD8^+^ T cells%**^#^** | 124 | 0.78(0.52,1.26) | 0.69(0.45,1.07) | 0.10 |
| CD4^-^CD8^-^ T cells%**^#^** | 124 | 1.68(0.67,3.28) | 0.99(0.89,1.11) | 0.91 |
| NK cells%**^#^** | 124 | 18.5(11.5,28.1) | 1.00(0.99,1.00) | 0.25 |
| T4/T8 ratio%**^#^** | 124 | 1.65(1.13,2.19) | 1.04(0.77,1.42) | 0.78 |
| B lymphocytes%**^#^** | 124 | 11.6(7.51,16.5) | 1.00(0.96,1.04) | 0.96 |

Univariate Cox regression models were used. N, number of participants; HR, hazard ratio; CI, confidence interval; NK cells, natural killer cells.

**Table S12.** Adjusted cox regression model to predict the time to hospital re‑admission

| **Variables** | **B** | ***P*** | **Adjusted HR** | **95% C.I. for HR** | |
| --- | --- | --- | --- | --- | --- |
|  |  |  |  | **Min** | **Max** |
| Home oxygen therapy | 0.666 | 0.03 | 1.95 | 1.06 | 3.57 |
| SGRQ score | -0.001 | 0.99 | 1.00 | 0.98 | 1.03 |
| mMRC score | 0.087 | 0.75 | 1.09 | 0.64 | 1.85 |
| exercise capacity score | 0.009 | 0.98 | 1.01 | 0.50 | 2.05 |
| CAT score | 0.008 | 0.84 | 1.01 | 0.94 | 1.08 |
| COPD-related exacerbation within the previous year | 0.317 | 0.01 | 1.37 | 1.08 | 1.74 |

Cox regression models were used. N, number of participants; HR, hazard ratio; CI, confidence interval; Max, maximum; Min, minimum.
